# Perspectives of 360-Degree Cinematic Virtual Reality: Interview Study Among Health Care Professionals

**DOI:** 10.2196/32657

**Published:** 2022-04-29

**Authors:** Elizabeth Beverly, Brooke Rigot, Carrie Love, Matt Love

**Affiliations:** 1 Ohio University Heritage College of Osteopathic Medicine Athens, OH United States; 2 J Warren McClure School of Emerging Communication Technologies Ohio University Athens, OH United States

**Keywords:** virtual reality, qualitative, medical education, health care, digital learning, learning platform, health care providers

## Abstract

**Background:**

The global market for medical education is projected to increase exponentially over the next 5 years. A mode of delivery expected to drive the growth of this market is virtual reality (VR). VR simulates real-world objects, events, locations, and interactions in 3D multimedia sensory environments. It has been used successfully in medical education for surgical training, learning anatomy, and advancing drug discovery. New VR research has been used to simulate role-playing and clinical encounters; however, most of this research has been conducted with health professions students and not current health care professionals. Thus, more research is needed to explore how health care professionals experience VR with role-playing and clinical encounters.

**Objective:**

The aim of this study was to explore health care professionals’ experiences with a cinematic VR (cine-VR) training program focused on role-playing and clinical encounters addressing social determinants of health, Appalachian culture, and diabetes. Cine-VR leverages 360-degree video with the narrative storytelling of cinema to create an engaging educational experience.

**Methods:**

We conducted in-depth telephone interviews with health care professionals who participated in the cine-VR training. The interviews were audio recorded and transcribed verbatim. A multidisciplinary team coded and analyzed the data using content and thematic analyses with NVivo software.

**Results:**

We conducted 24 in-depth interviews with health care professionals (age=45.3, SD 11.3, years; n=16, 67%, women; n=22, 92%, White; and n=4, 17%, physicians) to explore their experiences with the cine-VR training. Qualitative analysis revealed five themes: immersed in the virtual world: seeing a 360-degree sphere allowed participants to immerse themselves in the virtual world; facilitated knowledge acquisition: all the participants accurately recalled the culture of Appalachia and listed the social determinants of health presented in the training; empathized with multiple perspectives: the cine-VR provided a glimpse into the real life of the main character, and participants described thinking about, feeling, and empathizing with the character’s frustrations and disappointments; perceived ease of use of cine-VR: 96% (23/24) of the participants described the cine-VR as easy to use, and they liked the 360-degree movement, image resolution, and sound quality but noted limitations with the buttons on the headsets and risk for motion sickness; and perceived utility of cine-VR as a teaching tool: participants described cine-VR as an effective teaching tool because it activated visual and affective learning for them.

**Conclusions:**

Participants emphasized the realism of the cine-VR training program. They attributed the utility of the cine-VR to visual learning in conjunction with the emotional connection to the VR characters. Furthermore, participants reported that the cine-VR increased their empathy for people. More research is needed to confirm an association between the level of immersion and empathy in cine-VR training for health care professionals.

## Introduction

### Background

The global market for medical education is expected to grow by US $143.3 billion during 2021-2025 [1]. Although numerous factors will contribute to this rise, new modes of delivery are expected to be a major driving factor [1]. Virtual reality (VR) is one mode of delivery propelling the growth of the medical education market [2]. In 2019, the global VR health care market was valued at US $2.1 billion [3]. By 2026, it is expected to reach US $30.4 billion, with a compound annual growth rate of 42.4%. Advances in VR technology and improved accessibility and affordability of wearable devices are driving the demand for VR in medical education [2]. Moreover, the COVID-19 pandemic emphasized the need for virtual learning opportunities in medical education [4].

VR is technology that simulates real-world objects, events, locations, and interactions in 3D multimedia sensory environments [5,6]. Users explore sensory environments in real time (ie, first-person active learning) through different levels of immersion [6]. Levels of immersion range from low to moderate to high, depending on the degree to which the VR is inclusive, extensive, surrounding, vivid, and matching [7]. Inclusive refers to whether the VR removes signals (eg, external noise) from the physical world. Extensive refers to the number of senses engaged in the VR. Surrounding refers to the presentation of the VR and the degree to which the physical world is blocked out. Vivid refers to the fidelity and resolution of the VR. Matching refers to whether the VR is adapted to fit proprioceptive feedback from, or spatial awareness of, the user. Fully immersive VR reaches high levels of inclusive, extensive, surrounding, vivid, and matching in the virtual world such that the user experiences the virtual world as if it were the real world [2]. An example of fully immersive VR is viewing 360-degree video using a headset with controllers so that the user can see, hear, and move in, as well as interact with, the virtual world. In contrast, semi-immersive VR provides a user with a virtual environment that is not fully inclusive, extensive, surrounding, vivid, or matching [2]. It provides a user with a complete picture of the virtual world; however, the user is limited in their ability to move in, or interact with, this virtual world [2]. In other words, the user has a strong connection to the virtual world, but they are not completely isolated from the real world. An example of semi-immersive VR is viewing a 360-degree video on a web-based or smart device–based platform.

### Objectives

Traditionally, VR in medical education has been used to supplement surgical training [8,9], teach human anatomy [10], and visualize molecular complexes to advance drug discovery [11]. More recently, VR has been used to simulate clinical skills training and role-playing [12,13]. VR offers the user repeated attempts to practice difficult conversations or scenarios, with fewer time constraints and less perceived pressure from observation by superiors [12,14]. Research has shown that VR with role-playing and clinically based scenarios enhances empathy toward patients in medically and culturally diverse populations [14-16]. Most of this research has focused on health professions students and not professionals, although a new study demonstrated the effectiveness of improving cultural self-efficacy and attitudes toward diabetes among health care professionals [17]. Thus, more research is needed to understand how VR with role-playing and clinical encounters can be used effectively for practicing health care professionals. The aim of this study was to explore health care professionals’ experiences with a cinematic VR (cine-VR) training program. Specifically, this qualitative study assessed the educational impact and knowledge learned among health care professionals after the cine-VR training.

## Methods

### Research Design

Cine-VR is close to a fully immersive experience. Users have the ability to look around the virtual world in 360 degrees and hear sounds with spatialized audio, thereby creating a highly inclusive, surrounding, and vivid experience. This experience reinforces the belief that the user is present in the virtual world. Cine-VR differs from traditional VR, which uses computer-generated characters and worlds; in contrast, cine-VR uses live images filmed through a camera such as in cinema. In cine-VR, filmmakers leverage 360-degree video and apply the techniques of cinema to VR. Techniques include narrative storytelling, scripts, actors, lighting, framing, lens choices, focus, color, and so on. These techniques create an engaging educational experience for users. Studies have shown that immersion technologies such as cine-VR enhance education by allowing multiple perspectives, situated learning, and improved knowledge transfer to other situations [17,18].

We used narrative inquiry to understand health care professionals’ experiences with the cine-VR technology and training program [19]. For the purposes of this study, health care professionals included physicians, nurse practitioners, nurses, psychologists, exercise physiologists, physical therapists, dietitians, pharmacists, certified diabetes educators, community health workers, and certified health education specialists. Narrative inquiry captures the relationship between the individual experience and the greater cultural context through the communication of knowledge and the experience of time [19]. In this study, narrative inquiry described the lived experience of participating in an innovative cine-VR training program for continuing medical education and continuing education credits in the evolving landscape of medical education.

### Cine-VR Training

We conducted in-depth telephone interviews with a subset of health care professionals who participated in the 3-hour cine-VR training program entitled *Using Virtual Reality to Visualize Diabetes in Appalachia*. The purpose of this cine-VR training program was to educate health care professionals in Ohio about implicit bias and social determinants of health per the funding announcement. We selected education to address bias toward Appalachian culture, social determinants of health, and type 2 diabetes, considering that more than one-third of the counties in Ohio are Appalachian and their diabetes rates are nearly double the national average (20% vs 11%) [20]. The participating professionals watched 10 cine-VR simulations and 2 traditional films that displayed interactions between a woman aged 72 years with type 2 diabetes and her primary care physician, pharmacist, family members, and community. The cine-VR featured 4 *guided simulations*, which were face-to-face role-playing conversations with the main character and her providers. All cine-VR simulations were screened in an Oculus Go (Reality Labs) head-mounted display to allow participants to turn their head and body in any direction to gather information as though they were present in the actual location. In addition to the cine-VR simulations, we delivered 12 brief didactics (eg, 3 to 5 minutes for each didactic) that addressed the following content: (1) diabetes burnout, (2) food insecurity, (3) Appalachian cultural strengths, (4) transportation barriers in rural areas, (5) person-centered care, (6) psychosocial issues in diabetes, (7) financial insecurity and the cost of diabetes medications, (8) lack of social support, (9) Appalachian cultural values, (10) diabetes complications, (11) diabetes comorbidities, and (12) effective communication. The participating health care professionals received 3.0 continuing medical education or continuing education credits at no cost. The primary aim of the original study was to improve participants’ cultural self-efficacy and diabetes attitudes, wherein we observed statistically significant improvements in all subscales of cultural self-efficacy and diabetes attitudes after training; a detailed description of these findings has been published elsewhere [17].

### Cine-VR Technology

A variety of cameras were used to create the cine-VR images. Specifically, we used Insta360 OneX and Insta360 Pro 2 with a Sennheiser Ambeo soundbar and Zoom F6 microphones. For most of the filming, we used the Insta360 Pro 2, which allowed us to capture the entire 360-degree space at the same time. This was achieved with multiple sensor-lens combinations that capture different portions of the image simultaneously. These disparate images were combined into a single 360-degree panorama, either in real time before the image was saved to the memory card or later using Final Cut Pro (Apple Inc) and DaVinci Resolve (Blackmagic Design).

### Ethics Approval

Ethics approval for the study was obtained from the Ohio University Office of Research Compliance Institutional Review Board (20-X-111). In complying with federal, state, and local laws and regulations for human subjects research, we ensured that our research met the requirements set forth in the regulations on public welfare in part 46 of title 45 of the Code of Federal Regulations, the principles set forth in the *Belmont Report*, and the Helsinki Declaration of 1975.

### Sample

We used maximum variation sampling, a form of purposive sampling [21], to recruit a wide range of participants from different disciplines. The inclusion criteria for participation included adults aged ≥18 years who could read and speak in English and were currently employed as health care professionals (ie, physicians, nurse practitioners, nurses, psychologists, exercise physiologists, physical therapists, dietitians, pharmacists, certified diabetes educators, community health workers, and certified health education specialists) in Ohio. Participants were recruited through email and word of mouth. Specifically, we emailed participants who shared their email addresses with us after participating in the training or we spoke with individuals who completed the training.

### Data Collection

All interviews were conducted by two trained qualitative researchers (EB and CL), who asked participants broad, open-ended questions about the cine-VR content and educational experience. Specifically, participants were asked what they learned about diabetes, social determinants of health, and Appalachian culture during the cine-VR training program. The researchers used directive probes to clarify questions and elicit additional information from the participants (Textbox 1). All interviews were conducted through telephone because of the COVID-19 pandemic and restrictions with in-person human subjects research because of state-mandated lockdowns. The interviews ranged from 20 to 45 minutes in length. We collected data until we achieved data saturation; that is, when variation in the data leveled off or no new perspectives emerged from the interviews [21]. All interviews were digitally audio recorded and transcribed verbatim. The researchers performed quality checks of the transcribed files by listening to half of the interview recordings to validate the data. The transcripts were cleaned to remove participants’ names and identifiers to protect confidentiality.

**Interview questions.**

**Questions**

- What is your clinical, teaching, or academic role?
- Overall, how would you describe your experience with the virtual reality?
- In your own words, how would you have described Appalachian culture before you watched the virtual reality?
- Did your view of Appalachia change after seeing the virtual reality? Please explain.
- After watching the virtual reality, how do you believe diabetes is affecting Appalachian Ohio?
- In your opinion, how is diabetes viewed in rural Appalachia?
- Prior to the virtual reality, what did you think were the biggest barriers to diabetes care in Appalachian Ohio?
- After watching the virtual reality, what are the biggest barriers to diabetes care in Appalachian Ohio?
- What was the most important factual information you gained as part of this experience?
- What aspect of this experience had the most impact on your attitudes and beliefs about this people with diabetes in Appalachian Ohio?
- How will this experience most impact your future behavior when working with people in Appalachian Ohio?
- How does virtual reality compare to the different learning styles you have experienced in your career?
- In your opinion, what makes the virtual reality effective or ineffective?
- What suggestions or recommendations do you have for improving the virtual reality experience?
- Is there anything else about this virtual reality experience that you want to share?

### Qualitative Analysis

The multidisciplinary research team, which consisted of a behavioral diabetes researcher, a medical student, and 2 VR experts, used standard qualitative techniques to analyze the data. First, two members of the research team (EB and BR) performed content analysis by independently coding common words, phrases, and ideas in the qualitative data [22-24]. They met to review coded data to establish intercoder reliability; all discrepancies were reviewed, discussed, and resolved through consensus [24]. The Cohen κ coefficient between the 2 coders was 0.951, indicating almost perfect agreement [25,26]. No negative cases were excluded from the analysis [27]. After all the transcripts were coded and reviewed, one member of the research team (BR) entered the coded transcripts in NVivo software (QSR International) to organize the coded data. The two remaining members of the team (CL and ML) reviewed the codes to achieve researcher corroboration. The research team selected themes that characterized the participants’ experiences with the cine-VR technology and training program that occurred multiple times, both within and across transcripts.

### Rigor

We used investigator triangulation with team members representing different disciplines to support the credibility (ie, validity) of the data. Investigator triangulation also provided a means to identify cognitive biases in the analysis [28]. We reviewed findings with 21% (5/24) of the participants to achieve participant corroboration and establish validity of the accounts [29]. Next, we supported transferability (ie, external validity) through rich descriptions of the participants’ experiences with the cine-VR training program and the inclusion of verbatim quotations [27]. To support dependability (ie, reliability), we invited a researcher, Marilyn D Ritholz, not involved with the study to conduct an inquiry audit to examine the process and the product to determine whether the findings and conclusions were supported by the data [27]. Finally, to establish confirmability (ie, objectivity), we created an audit trail to document the research steps we took from the start of the study to the reporting of the findings [27].

## Results

### Overview

A total of 24 health care professionals participated in in-depth telephone interviews; the mean age of the participants was 45.3 (SD 11.3) years; 18 (75%) identified as women, 4 (17%) as men, 1 (4%) as gender queer, and 1 (4%) as transgender or nonbinary. Of the 24 participants, 22 (92%) self-identified as White and 2 (8%) as Asian (Table 1); 5 (21%) were community health workers or certified health education specialists, 4 (17%) were physicians, 4 (17%) were nurses, 3 (13%) were dietitians (13%), 2 (8%) were pharmacists, 2 (8%) were exercise physiologists, 2 (8%) were medical directors, 1 (4%) was a physical therapist, and 1 (4%) was a psychologist. Years of clinical or academic experience ranged from 1 to 5 years (7/24, 29%), 6 to 10 years (4/24, 17%), 16 to 20 years (7/24, 29%), 21 to 25 years (4/24, 17%), and >25 years (2/24, 8%).

**Table 1:** Participants’ demographic characteristics (N=24).

Characteristic		Values
Age (years), mean (SD)		45.3 (11.3)
**Gender, n (%)**		
	Woman	18 (75)
	Man	4 (17)
	Gender queer	1 (4)
	Transgender or nonbinary	1 (4)
**Race, n (%)**		
	American Indian or Alaska Native	0 (0)
	Asian	2 (8)
	Black or African American	0 (0)
	Hispanic or Latinx	0 (0)
	Native Hawaiian or Pacific Islander	0 (0)
	White	22 (92)
	Two or more races	0 (0)
	Another race not listed	0 (0)
**Occupation, n (%)**		
	Community health worker or certified health education specialist	5 (21)
	Physician	4 (17)
	Nurse	4 (17)
	Dietitian	3 (13)
	Pharmacist	2 (8)
	Exercise physiologist	2 (8)
	Medical directors	2 (8)
	Physical therapist	1 (4)
	Psychologist	1 (4)
**Years in health care, n (%)**		
	1 to 5	7 (29)
	6 to 10	4 (17)
	11 to 15	0 (0)
	16 to 20	7 (29)
	21 to 25	4 (17)
	>25	2 (8)

Transcript identifiers are included with quotations indicating participant number and discipline. The following themes emerged from the data analysis:

### Immersed in the Virtual World

Of the 24 participants, 22 (92%) described feeling immersed or connected to the virtual world. They commented on the realism they felt in the cine-VR simulations. Participants identified with the dress, the cars, the houses, the people, and the barriers they faced:

I identified with the video. I identified with the dress. The vehicles. The scenarios and problems that those families face. The coexisting disorders. The housing. How things are undone and unfinished. Raising grandchildren. You know, taking care of everyone else but yourself, and being viewed and stereotyped as someone who is heavy and lazy, and maybe just doesn’t care when really that is not the case in impoverished areas such as southeast Ohio.

ID 103, Nurse

Just for me, [it was] the realism of it. It’s our neighbors. It’s what we see here in Appalachia. It’s the patients that I’ve dealt with since 1994 when I became a nurse. So I think it was well done. I think the realism was really important and impactful for a lot of folks who aren’t from here.

ID 101, Nurse

**Figure 1:**
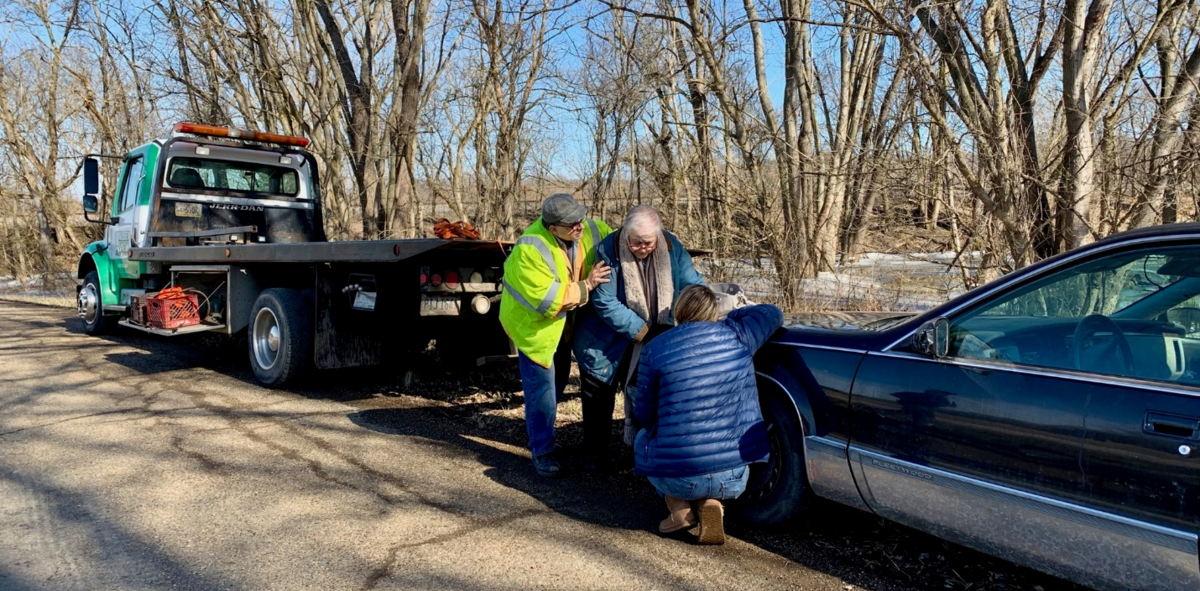
Photograph of the main character, her daughter, and a tow truck driver. The main character’s car breaks down on the side of a rural road. Photograph by ML.

The cine-VR gave participants privileged, unfiltered access to the main character’s environment, social interactions, and daily challenges. In particular, the cine-VR slice-of-life vignettes depicted the main character’s struggles with social determinants of health, including poverty, food insecurity, housing instability, transportation barriers, lack of access to health care, and limited social support. Seeing a 360-degree sphere allowed participants to immerse themselves in the simulations, feeling the disarray and chaos of the main character’s home or the substantial burden of the family car breaking down (Figure 1):

The first time I watched it I was just in awe. I felt like I was a fly on the wall. I can look all around. I can look at the unfinished walls. I can look at the clutter. I can look at the unwashed dishes that might been two or three days, maybe an overflowed trashcan. Kids in and out. The chaos, the child with the traumatic brain injury that’s just kind of part of the wallpaper. I mean, they’re existing in there and we’re all working our lives around them. I think that happens all the time in rural Appalachia. But to be inside someone’s home and inside their life, it’s almost like being invisible and seeing all the things that people want to hide either purposely or indirectly that affect their life. It’s like a glimpse inside someone’s personal life and why things are the way they are.

ID 103, Nurse

I think that the whole scene with the flat tire with the car I think was huge because it wasn’t just the flat tire, right? There was the flat tire, there was the fact that then she didn’t have money to pay the tow truck driver and she was going to thank him with a cake because that’s how she shows her caring for people is through baking sweets. I think all of that happens all the time...There’s just so much...I felt like that scene stacked everything up really nicely. This could 100% happen to anyone on any given day because it felt very realistic.

ID 112, Medical director

These participants viewed the main character, her environment, and her interactions with professionals as true to life and clinical practice in Appalachian Ohio. In fact, some participants felt that the cine-VR was so realistic and immersive that the main character must be a real person or one of their patients:

So I knew it was simulated, right? It's stuff I see every day. But I think what I liked about it is it solidified all the things I thought. I’m like, “Well, I don’t think people can get in their car and go, I think people smoke, and I think they have a hard time taking care of their families.” It was like taking all the research that everybody’s done and putting it into an actuality that actually is actual. I still think about it almost like it was my patient, right? I remember thinking of her in the car and twisting her [ribbon] around her finger, and her husband dying. I remember all of it like she was one of my patients.

ID 109, Pharmacist

Furthermore, the realism of the cine-VR left a lasting impression on the participants. Several of the participants stated that they look back on the simulations to inform their current clinical practice:

It was engaging. It’s very realistic. It resonated well with what I can envision my patients dealing with and brought to light a lot of the issues that I don’t think a lot of physicians can envision well without seeing something like this...To see it come to life in that format really made it real, and helps it stick so I remember that when I’m dealing with patients every day. And it’s probably not exaggerating to say that I think about what I saw in the Lula Mae videos when I’m taking care of patients in the emergency department. And I think it’s probably been a year or so since I’ve looked at the videos and I’m still flashing little scenes of what I saw in those videos when I’m interacting with patients, so it’s powerful. Realistic. Really hit the mark to bring a lot of things to light.

ID 123, Physician

### Facilitated Knowledge Acquisition

All the participants accurately recalled the culture of rural Appalachian Ohio and listed the social determinants of health presented in the cine-VR training program. As all interviews were conducted 12 to 18 months after the training program, this suggested that the participants successfully learned and retained the information. They described the cultural strengths of Appalachia, including caregiving, loyalty, and generosity, as follows:

I would describe the people as close-knit communities...Everyone knows each other. Everybody helps each other out for the most part. If someone needs something, people dig deep, and give ’til it hurts when they don’t have much to give at all.

ID 103, Nurse

I would describe Appalachian culture as a passionate group of people, people who love their families and those that they claim as family, which may or may not be blood-related. I would describe them as very self-reliant.

ID 104, Nurse

I think you really hit it with that, especially because she’s [Lula Mae] clearly the matriarch in that situation. How many family units do we see in Athens County and surrounding counties that are based on that matriarch, and that’s huge. I think that’s a really positive thing.

ID 112, Medical director

Participants also remembered the numerous social determinants of health affecting people in this rural and underserved area. Participants identified financial insecurity, food insecurity, transportation barriers, lack of access to health care, low educational achievement, and social isolation as social determinants of health negatively affecting people living in rural Appalachian Ohio:

There are many social factors that are barriers. It’s not just the health care infrastructure that’s the problem. It’s all of the other life stressors and challenges that make it difficult to prioritize a person’s individual health. Poverty, food insecurities, transportation barriers.

ID 117, Certified health education specialist

Money. Not only access to care, but money to purchase the things needed. Education to understand how to take care of yourself. How to monitor your own glucose, et cetera.

ID 102, Psychologist

I would say transportation for sure. Maybe not that the person doesn’t have a car; they might have a car, but maybe they don’t have money to put the gas in the car. Or, maybe it’s not reliable enough to get them where it is that they need to go.

ID 104, Nurse

I think one of the things that really are important to harp on are the isolation that a lot of people feel here.

ID 111, Dietitian

Multiple participants noted how low educational achievement in Appalachian Ohio was a barrier to health care, citing both a lack of education regarding one’s personal health as well as lack of education regarding the severity of diabetes:

I feel like we are still very undereducated about diabetes in this area. I don’t think people understand the impact that it makes. I don’t think the people living here actually understand the impact that it makes on their health. I feel like—I guess I said it in my last statement when I said it’s not a priority. I don’t feel that people in this area feel like their health is a priority.

ID 109, Pharmacist

I think it really comes down to their lack of education and their lack of understanding not only to their predisposition for it, their risk of it, but how serious it is once you are, in fact, diagnosed with it and how you care for yourself.

ID 102, Psychologist

In addition, many participants discussed common beliefs about diabetes in Appalachian Ohio. They described the sense of fatalism surrounding a diabetes diagnosis because of the high prevalence of the disease in the area. Participants thought that this fatalist attitude toward diabetes prevented people from making behavior changes to prevent or delay the onset of diabetes:

I think that there is some, definitely some folks who think, “Well everyone in my family’s had it so I’m going to get it too,” because that’s what they’ve seen and that’s what they know.

ID 104, Nurse

I think most just are—I’m trying to think of the word—resigned to the fact that it’s going to happen...If somebody else in their family has had it, they assume they’re going to get it.

ID 101, Nurse

### Empathized With Multiple Perspectives

**Figure 2:**
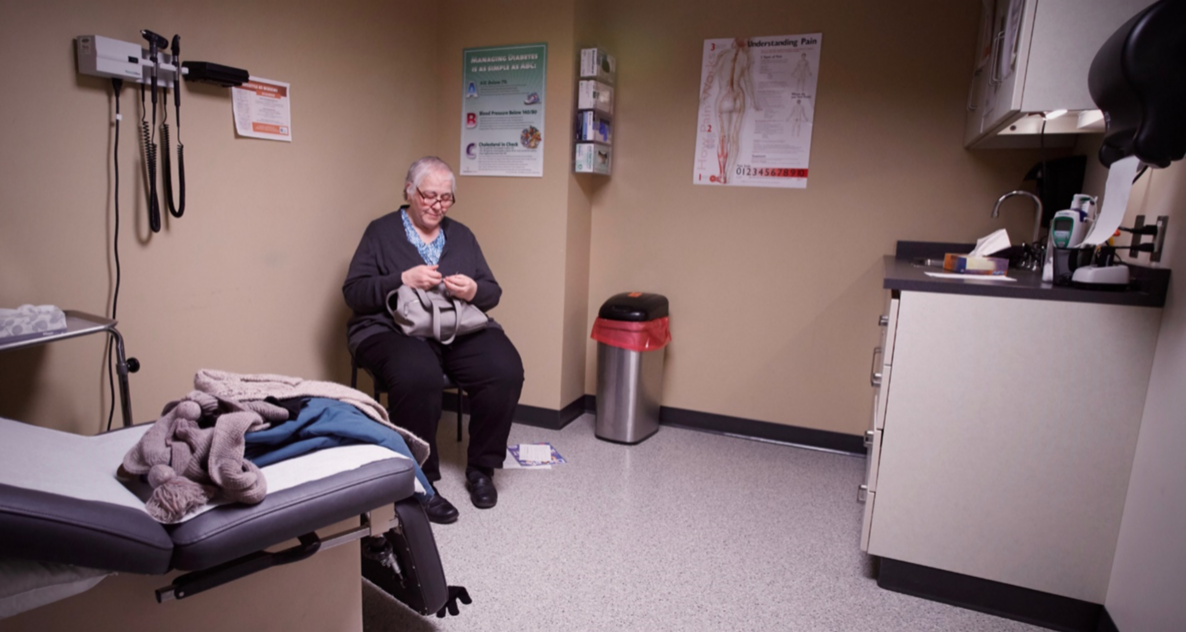
Throughout the cinematic virtual reality, the viewers watch the main character take care of everyone in her life and as a result put her health and well-being last. This photograph of the main character depicts the isolation and lack of support she receives in her diabetes management. Photograph by ML.

Of the 24 participants, 21 (88%) commented on the empathy they experienced during the cine-VR simulations. The cine-VR provided a glimpse into the real life of the main character, and participants could sense what she was thinking and feeling. Perceiving these thoughts and feelings may have transferred the experiences to the users. Participants described feeling Lula Mae’s frustrations, her disappointments, and “being torn between competing responsibilities” (Figure 2):

You really got to see life through her—I felt like I was walking in her shoes? I felt emotions as part of that learning experience. I felt the frustrations that she might’ve felt. The disappointments, and being torn between competing responsibilities. I felt like I understood why she wasn’t taking her medication as prescribed, and it didn’t seem like it had anything to do with her desire to address her own health, but more a function of her taking care of everyone else and all of the responsibilities that came with that. She kept putting herself last. I think it’s a realistic reflection of what many people do when they’re overburdened and overstressed and underresourced.

ID 117, Certified health education specialist

It’s the emotions, yes. When it feels like you’re right there observing Lula Mae in her environment, and her interactions with the family, with the doctor, with the community members, you can almost feel her or the other people’s emotions. Like when Lula Mae was stuck on the road, I could feel her desperation because, hey, I was stuck on the road once, and I did not like it at all. You’re empathizing with her. When she was at the doctor’s office, I could feel her anxiety.

ID 107, Medical director

Participants also reflected on the thoughts and feelings of the other characters in the cine-VR. They recognized that identifying harmful emotions from the professionals and family members was as important as empathizing with the main character. They explained that understanding the perspectives of each of the characters helped them to identify how they could support people with diabetes in the future:

The way that it makes you internally reflect on how that situation could apply to you or how you could possibly be as a provider or as an educator in the shoes to help someone like Lula Mae. Because I would say she just felt beat down and almost helpless—one thing after another. It made me reflect and it made me think like if I were in even the nurse’s shoes at the doctor’s office or the medical assistant at the doctor’s office, if I were in that person’s shoes, what could I do? Or her family support was not there. She was the support for the whole family. So, as a family member, how can you be there for people, or as a friend, how can you be there for people so that you don’t have a friend that feels like they’re in Lula Mae’s situation? Because I think that’s what it made me look at is I never want one of my friends who has diabetes to feel like that and like they’re alone and try to figure it out all on their own. So, I think one of the best things it gives you is it truly gives you a feeling for what it’s like, and it gives you that time to reflect and discuss what’s going on.

ID 113, Exercise physiologist

Looking at it from different perspectives—it was more impactful that way. Because you got to see and kind of empathize, if you will, just how it was coming from the perspective of that patient who maybe didn’t have the money for medication, or from the doctor’s perspective, being so overwhelmed and so tired in the health care system he’s working in right now. Or the feeling of you’ve got a patient who has so many roles to fulfill because of the plate that she has been handed. Like taking care of grandchildren or taking care of the family member that needed her. Then also the spiritual side of it where she was actually trying to help other people by doing what she felt was right at the food pantry. It was just wholehearted.

ID 119, Nurse

Some of the participants expressed an intention to change how they practiced medicine after viewing the simulations. They explained that it increased their empathy for patients and enhanced their understanding of the challenges faced by people in rural Appalachia:

It changed my practice in the sense it made me a heck of a lot more empathetic towards what they may or may not be saying. I always look at body language and can read that pretty well, but now I understand a little bit of what might be going on. It also helped me to understand the culture of my patient population and what things may be important, not important to them, and what may be playing a role in why they’re making the decisions that they’re making or making the decisions where they’re not able to take care of their health because they are so burdened with so many issues in their lives.

ID 122, Physician

### Perceived Ease of Use of the Technology

Of the 24 participants, 5 (21%) had prior experience with VR technology; however, none of them had prior experience of cine-VR. When asked about its ease of use, 96% (23/24) of the participants described the cine-VR as easy or simpler than expected:

The technology was pretty simple actually. I mean, when we had somebody who was facilitating, who was very understanding of the technology, it was very easy...Clinicians are already using technology on a daily basis to do their charts and things so this is much less burdensome than [electronic health record system] so to speak. So as far as I’m concerned, no, this is really easy, very simple.

ID 122, Physician

It was a lot easier than I thought it would be, to be honest with you. Because aside from a couple of experiences using the headsets, I’m not a gamer and I don’t use them for other things like people who do are used to that environment. But from picking it out of the box to turning it on, to putting it on my head, getting the right video, and getting it going was remarkably easy.

ID 123, Physician

Several participants reported feeling surprised by the live-action VR. They did not know it was possible to see live-action VR using a headset:

Live action virtual reality is what really grabbed me about this one. I’ve seen a lot of virtual reality avatar-based things and a lot of things where you’re grabbing the virtual syringe and you’re injecting the virtual syringe into the virtual type patient. It’s okay. I think we’re still probably like years off for where that really has the virtual tactile feel that I need to make a valuable trend. Where this one really hit was that it used live action where live action was the best way to do it. So, if I really want to feel what it’s like to be in you know, Lula Mae’s house, you know, then boy, this is the way to do it.

ID 123, Physician

Participants also commented on the ability to move 360 degrees in any direction during the simulation as well as the quality of the image and sound:

It was more than I expected. I was—I guess I was not expecting the 360-degree virtual reality and to have so much freedom with movement to be able to look all around me, around the room. That type of technology was pretty groundbreaking to me. I was more expecting to just look straight ahead and everybody would be seeing the same thing at the same time.

ID 106, Dietitian

I really liked the video and the sound quality, especially. My hearing isn’t always the best. I was in marching band, and I played drum line, and I work at a really busy pharmacy. So being able to hear so clearly with that technology was really, really engaging. And then the video quality, I was thoroughly shocked by. I was expecting more like my other experience of virtual reality, where it’s a little grainier.

ID 108, Pharmacist

The cine-VR training program had some technical and sensory issues. A few participants remembered accidentally hitting the buttons on the side of the headset, which turned off the simulations:

I think having a little presession, here’s how you use the Oculus or don’t press this button, because I accidentally turned it off twice, are important reminders. I am a technically savvy individual. However, even I struggled with it, despite the best efforts of all the support people there.

ID 124, Physician

The other main issue was worries about motion sickness (to address the potential dizziness and nausea from viewing the cine-VR, we disclosed that these risks were possible and offered alternative viewing devices). Of the 24 participants, 2 (8%) reported experiencing motion sickness and 3 (13%) thought that they would experience motion sickness but did not:

As far as the motion sickness that I personally experienced, a disclosure to let people know that there are bright lights flashing. The refresh rate of whatever the Oculus is may impair some folks and to be aware of that going in so that I didn’t, I wouldn’t be as sick, but that’s me personally. Not everyone may experience that.

ID 124, Physician

So, it was easier to use than I had anticipated it to be. I thought it was going to be kind of complicated. I also was a little worried about it making me like motion sick, because sometimes movies, like 3D movies will do that, but it wasn’t, none of that happened at all. It was super easy to use, easy to focus on things, and really neat to be able to look around the whole room and really get an entire picture of what’s going on in different parts of the house for example during different things. So, I would say all in all, it was completely positive, a very awesome experience and a very neat and different way to learn things, but it kept me engaged as well.

ID 113, Exercise physiologist

### Perceived Utility of Cine-VR as a Teaching Tool

Of the 24 participants, 22 (92%) described cine-VR as an effective teaching tool and 23 (96%) expressed satisfaction with the learning experience. The cine-VR training was viewed favorably by participants both with and without prior experience with VR technology:

I thought it was super cool. I’m not a gamer. I mean, I’m not a major tech person, so it was a really cool experience for me. I’d never done anything like that, and I haven’t done anything or seen anything like that since. So, if that’s the way things are headed, I think it’s pretty cool and it’s certainly a great way for the next generation to learn things.

ID 101, Nurse

Participants believed that the realism of the cine-VR offered a way of learning that affected the user on a personal level:

I think, especially as a clinician, I think you can go through training and training, you could read books, you can watch videos, you can look at PowerPoints, but when you’re forced to be in the room with someone like that and they’re looking right at you, and you look around and you’re the only one there, or—you know what I mean, you kind of have to be a little bit more involved so you have to be more attentive to what’s going on so you respond better.

ID 105, Exercise physiologist

Participants recognized the applicability of cine-VR as an educational tool for multiple topic areas and levels of learning. They felt that the realism of the simulations and full immersion in the virtual environment made the training an effective tool. Moreover, they believed that the cine-VR tapped into affective learning. Participants believed that visual learning in conjunction with emotional connection to the VR characters made the training program a meaningful and memorable experience:

This format is our first experience doing this, and I think aside from being realistic in the stories and the content resonating, the format really immersed the learner in the environment. We strive for realism. We strive for full immersion. We strive for really activating all of the senses to create the best learning environment. When you get all the senses firing and you immerse somebody fully into this reality, it creates memories and you feed into that psychology in different parts. Because it activates your emotional centers and visual cortex and your auditory cortex and all of this works together to really form all these connections for you to remember or bring into our practice. So yeah, but the full immersion, the application in this area, that’s what really has the power.

ID 123, Physician

Students nowadays are learning a completely different curriculum and a very case-based format. This is taking case-based formats to a whole new level because it’s not on paper, you are visually seeing it. And many people do better. Yes, they can do paper, but to actually see it almost like a movie. To almost embed it into your visual memory, it goes without saying that this is superior. There's no question about it...And I think that visual piece in the moment in the life, it’s just like a movie or a TV show where there are parts of it that you just take away and they embed, and you remember that and you can’t forget it in a way that you can totally forget a piece of paper with writing on it.

ID 122, Physician

## Discussion

### Principal Findings

In this qualitative study, we explored health care professionals’ experiences with a cine-VR training program in diabetes, social determinants of health, and Appalachian culture. Participants described feeling immersed in the cine-VR training. They commented on their ability to watch the main character interact with professionals, family members, and the environment by means of the cine-VR 360-degree video. They noted that the opportunity to see life through the main character’s eyes elicited many emotions in them. Most of the participants reported feeling the frustrations, anxieties, and disappointments experienced by the main character and empathizing with her struggles. Participants agreed that these emotions and empathy were key to the learning process and knowledge they acquired. All participants were able to recall the social determinants of health addressed in the cine-VR as well as the cultural aspects of rural Appalachia. Finally, 96% (23/24) of the participants described the cine-VR as easy to use with surprising technical features, including live action, 360-degree movement, high resolution, and high sound quality. Drawbacks included the buttons on the headset and risk for motion sickness. In summary, these findings suggest that health care professionals perceived immersion and empathy to be key drivers in the success of the cine-VR training.

### Comparison With Prior Work

The value of cine-VR training is that it gives health care professionals a glimpse inside the lives of their patients and why things are the way they are. The more professionals can understand their patients’ personal lives, the more they can empathize with their challenges and struggles. Recent gaming research found that immersive VR from the first-person perspective of a person with chronic pain increased kindness (ie, a subscale in the Empathy Scale) and willingness to help after a simulation, suggesting an increase in implicit and explicit empathy [30]. Another study on perspective-taking tasks showed that VR-based tasks increased self-reported empathy more than narrative-based tasks in a simulation about the homeless population [31]. These studies support the use of VR to promote empathy in medical education. Empathy in diabetes care is critical, given prior work demonstrating a 40% to 50% lower risk of all-cause mortality at 10-year follow-up when patients newly diagnosed with type 2 diabetes experienced high levels of provider empathy in the first year after diagnosis [32]. Participants in our study described the empathy they felt for the characters in the cine-VR. Thus, cine-VR training has the potential to increase empathy among health care professionals and students; however, more mixed methods research is needed to measure empathy and other prosocial behaviors (ie, helping, sharing, and comforting) before and after completing this cine-VR training.

Participants highlighted the importance of feeling immersed in the virtual world. A recent systematic review and meta-analysis by Kyaw et al [6] found that VR improved knowledge and skills acquisition among health care professionals compared with traditional modes of education (eg, textbooks and lectures) and other digital education (eg, 2D images on a screen and web-based teaching). The findings from this systematic review and meta-analysis suggest that VR is an effective mode of delivery for medical education and VR is more effective than traditional or other digital education in knowledge and skills acquisition [6]. Similar findings have been reported among medical students: those participating in fully immersive VR reported significantly higher gains in knowledge than students in partially immersive VR [33]. Another recent study focused on social determinants of health and empathy in dental education found that 360-degree, immersive VR improved cognitive, affective, and skills-based learning in residents and faculty [34]. Overall, these findings combined with the qualitative responses from our participants underscore the importance of immersive VR to enhance the user experience and improve learning outcomes.

### Limitations

The study limitations included homogeneity of the sample with regard to gender and race and ethnicity. Thus, the qualitative findings may not be transferable or generalizable to people not represented in the sample. Future research with a more diverse sample is necessary to explore experiences with the cine-VR training program. Additional limitations of the study included the small sample size and participant self-selection, which also limit the transferability of the findings. However, qualitative research differs from quantitative research in that it is not driven by sample size, randomness, and power calculations. Rather, qualitative research rests on the notion of data saturation or the point at which no new information is collected for data analysis. Therefore, sample size was not an indicator of rigor in our study. Historically, an adequate sample size for an in-depth individual interview study is 15 to 20 participants [21]. With regard to participant self-selection, individuals who volunteered to participate in the interviews may have had more positive experiences with the cine-VR training than participants who did not volunteer to participate in the study. In addition, we recruited participants through email and word of mouth, which increased our selection bias because participants were not randomly selected to participate in the interviews. Finally, self-reported data are vulnerable to social desirability bias. To minimize bias, the researchers informed participants that their responses were confidential and could not be linked back to their personal identity. Furthermore, the investigators emphasized the voluntary nature of participation and explicitly informed the participants that their responses had no bearing on their employment. Finally, our original study did not include a control group as a comparison. Research comparing cine-VR with a proper control condition is underway to examine the effectiveness of cine-VR in changing health care professionals’ knowledge, beliefs, and empathy.

### Conclusions

The participating health care professionals perceived the cine-VR training to be a valuable educational experience that generated empathy toward the VR characters. They attributed the value of the cine-VR to the immersive and realistic nature of the 360-degree virtual environment. Future research is needed to examine the impact of cine-VR training on quantitative measures of immersion, empathy, and prosocial behaviors among current professionals and health professions students. Cine-VR has the potential to play an integral role in clinical training as medical education expands to meet the growing need for virtual platforms.

## Abbreviations

cine-VR: cinematic virtual reality
VR: virtual reality
